# Binaural Acoustic Scene Classification Using Wavelet Scattering, Parallel Ensemble Classifiers and Nonlinear Fusion

**DOI:** 10.3390/s22041535

**Published:** 2022-02-16

**Authors:** Vahid Hajihashemi, Abdorreza Alavi Gharahbagh, Pedro Miguel Cruz, Marta Campos Ferreira, José J. M. Machado, João Manuel R. S. Tavares

**Affiliations:** 1 Faculdade de Engenharia, Universidade do Porto, Rua Dr. Roberto Frias, s/n, 4200-465 Porto, Portugal; hajihashemi.vahid@yahoo.com (V.H.); abalavi.gh@gmail.com (A.A.G.); mferreira@fe.up.pt (M.C.F.); 2 Bosch Security Systems S.A., EN109-Zona Industrial de Ovar, 3880-080 Ovar, Portugal; pedro.cruz4@pt.bosch.com; 3 Departamento de Engenharia Mecânica, Faculdade de Engenharia, Universidade do Porto, Rua Dr. Roberto Frias, s/n, 4200-465 Porto, Portugal; jjmm@fe.up.pt

**Keywords:** urban sounds classification, stereo signal, sound base intelligent system, machine learning, genetic algorithm

## Abstract

The analysis of ambient sounds can be very useful when developing sound base intelligent systems. Acoustic scene classification (ASC) is defined as identifying the area of a recorded sound or clip among some predefined scenes. ASC has huge potential to be used in urban sound event classification systems. This research presents a hybrid method that includes a novel mathematical fusion step which aims to tackle the challenges of ASC accuracy and adaptability of current state-of-the-art models. The proposed method uses a stereo signal, two ensemble classifiers (random subspace), and a novel mathematical fusion step. In the proposed method, a stable, invariant signal representation of the stereo signal is built using Wavelet Scattering Transform (WST). For each mono, i.e., left and right, channel, a different random subspace classifier is trained using WST. A novel mathematical formula for fusion step was developed, its parameters being found using a Genetic algorithm. The results on the DCASE 2017 dataset showed that the proposed method has higher classification accuracy (about 95%), pushing the boundaries of existing methods.

## 1. Introduction

The analysis of ambient sounds can be very useful when developing sound base intelligent systems. In the last few years, sound based intelligent systems have received a lot of attention in indoor and outdoor scenarios. Some possible applications of these systems are smart devices and phones, robotics, data archiving, surveillance, security systems, and hearing aids. Acoustic scene classification (ASC) is defined as identifying the area of a recorded sound or clip among some predefined scenes [1]. ASC is a subset of algorithms and systems for audio understanding by machine learning audio based algorithms, i.e., computer audition (CA).

Computer audition systems attempt to suggest intelligent algorithms to extract meaningful information from audio data [2]. ASC is a preprocessing step in some of these systems that attempt to identify the scene of audio data, e.g., airport, park and subway, just to name a few. In many CA applications, the background audio of real-time speech/music should be discarded in the preprocessing stage or used for environmental noise assessment [3]. ASC helps CA systems to limit the search area, select a denoising strategy and enhance overall accuracy. ASC systems have many challenges, one of them is the different type of inputs, since the quality of the microphones or audio sensors varies, the number of recorded audios from a scene varies, and the sensors can finally be mono (single channel) or stereo (dual channel) [3].

Another challenge is choosing the right inputs for the classifier, which is known as feature extraction and selection. Some of the features used in previous works include Mel frequency cepstral coefficients (MFCC), wavelet, constant-Q transform (CQT), and histograms of oriented gradients (HOG) [4]. An additional problem is to select the best classifier. The Gaussian mixture models (GMMs), hidden Markov models (HMM), support vector machines (SVMs), ensemble learning and deep learning methods, such as convolutional neural network (CNN), are some of the most common used ASC classifiers [5].

The main goal of all ASC systems is to achieve the best classification accuracy with the lowest required quality input, maximum processing speed, and minimum implementation complexity; however, intrinsically, these goals can be contradictory. Low quality input and fast classification typically decrease the classification accuracy. The hybrid and complex systems usually are more accurate, especially in real world applications. Based on the aforementioned, the main goal of this research was to achieve the best classification accuracy and, at the same time, to increase the classifier training speed.

Due to the stereophonic nature of the used audio recordings, two audio channels were used to train different probabilistic classifiers. The outputs of the studied classifiers were then used in a novel nonlinear mathematical formula, which was optimized by a genetic algorithm to achieve the best possible accuracy. The use of two channels to train different classifiers, and the proposed fusion scheme based on a nonlinear transformation to combine the output of the used classifiers in order to obtain the final decision, are the main novelties of this research. The following of this article is organized as follows: Section 2 presents the related background of ASC. Section 3 presents the proposed method and gives details of the used dataset. Section 4 and Section 5 present the results and their discussion, respectively. Finally, Section 6 presents the main conclusions of this research.

## 2. Related Work

ASC research has been focused on two main areas, i.e., feature extraction and classification schemes. The classification schemes were divided into ensemble based methods and other classification schemes, such as those based on deep learning, which can be used to highlight the difference between previous works.

### 2.1. Feature Extraction and Preprocessing

There are many audio features that can be used in ASC. Short-Term Fourier Transform (STFT) is one of the public features used in acoustic research. This kind of feature was used for scene classification [4,6,7] or in a preprocessing step such as in [8]. Usually, STFT is not used individually and its features are combined with other features, such as Mel Frequency Cepstral Coefficients (MFCC), Mel-frequency cepstrum (MFC) and log-Mel spectrogram instead. MFCCs are coefficients of MFC that independently or in combination with STFT and wavelet can be used for audio processing [9]. Mel-frequency cepstrum is the logarithmic power spectrum of the linear cosine transform of short-term audio signals in a nonlinear scale of frequency, usually known as Mel. In the Mel spectrogram, the frequencies are transformed to a nonlinear scale, similar to the human auditory system response, i.e., the Mel scale. On the other hand, a spectrogram is an image related to the spectrum of signal (audio) frequencies. Mel based features, such as log-Mel spectrogram, Mel-frequency cepstrum, MFCC, log-Mel delta, and delta–delta, are among the most commonly used features in ASC. For example, the Log-Mel spectrogram has been used in [8,10,11,12,13,14,15,16,17,18,19,20,21,22], with differences between parameters such as filter banks, STFT and windowing function.

In some research, log-Mel spectrogram is used after some processing or in combination with other features. For example, Alamir [23] used log-Mel spectrogram and wavelet scattering, Wu and Lee [24] used audio framing and log-Mel spectrogram, and Log-Mel spectrogram image after median filtering was used in [25]. Log-Mel spectrogram clustered by k means [26] and Log-Mel spectrogram and Gammatonegram (Gamma) [27,28] are other types of Mel based features that have been used in ASC. Some researchers showed that the first and second temporal derivatives of log-Mel spectrogram are good ASC descriptors [29,30,31,32,33]. These first and second log-Mel spectrogram derivatives are known as delta and delta–delta features. Log-Mel energies [4], log-Mel filter bank (LMFB) [34,35], log-Mel band energies and Single Frequency Filtering Cepstral Coefficients (SFFCC) [36] and MFCC and log-Mel filter bank [37] are other types of Mel-based features that have been used for ASC.

Lostanlen and Andén used wavelet scattering features for ASC [38]; this family of feature is comparable to MFCC. Raw audio data without any preprocessing have been used for ASC in [39,40].

### 2.2. Classification Schemes

#### 2.2.1. Ensemble Methods

Nguyen et al. [41] proposed a CNN ensemble ASC method for tasks of the DCASE 2018 challenge. The authors combined the output probabilities of CNNs as ensembles of CNNs to improve ASC accuracy. Jung et al. [42] proposed an ensemble model that extracts some audio features. The authors trained several deep neural networks (DNNs) in parallel, and the scores from DNN classifiers were applied to a score-level ensemble block to make the final result. Singh et al. [43] combined deep convolutional neural network (DCNN) scores in a score-level ensemble step and made the final output. Sakashita and Aono [44] trained nine neural networks and used their outputs as an input of an ensemble learning block in order to increase accuracy. Jiang et al. [11] used an ensemble learning method to make final decisions using the output of CNNs. Mars et al. [45] improved the result of a ASC system by including two distinct and light-weight architectures of CNNs, by using an ensemble of the output of CNNs. Huang et al. [46] used the ensemble of four different CNNs and improved the final result by about 4%. Wang et al. [47] used a CNN ensemble for voting of all scores of CNNs and improved the classification result. Ding et al. [48] applied a composed of two CNN and GMM scores to an ensemble system to enhance accuracy. Xu et al. [49] ensembled deep classifiers that trained using different features with the goal of using complementary information features.

Gao et al. [50] ensembled the output of trained CNN networks on different representations to boost classification performance. Wang et al. [51] trained 5-layer or 9-layer CNNs with average pooling using features conveying complementary information. The authors developed several ensemble methods to integrate the outputs of the CNNs such as random forests and extremely randomized trees. Other research, such as in [52,53,54], used ensemble learning to integrate different classifiers that trained using different features in order to increase ASC accuracy. Sarman and Sert [55] used two ensemble methods, mainly bagging and random forest, to overcome the imbalance problem of data with minimum computational cost. The authors classified violent scenes based on audio signal. Alamir proposed a hybrid method which includes a CNN and an ensemble classifier in a parallel form [23], the features used by the classifiers being different.

Based on the above review, one can conclude that many researchers used ensemble learning to integrate the result of a set of trained classifiers in a fusion or post-processing step in order to increase ASC accuracy. Only a few researchers used ensemble learning as a primary classification step. Based on our best knowledge, the results of this research are usually equal to or less than similar ASC classification methods, mainly, based on deep learning.

#### 2.2.2. Deep Learning Methods

ASC algorithms mostly use CNN based architectures in the classification step. For example, a Multi-task Conditional Atrous CNN (CAA-Net) is used in [2]. Liu et al. [4] used CNNs as main learners and an extra random forest method for final classification. Visual Geometry Group CNN (VGG net) has been suggested as a good architecture for ASC in some research [5,11,21]. Naranjo-Alcazar suggested a VGG-style CNN where convolutional blocks were replaced with residual squeeze-excitation blocks [5]. Jiang et al. used twelve VGG style CNNs in the first step of their method [11]. Another simplified VGGNet-InceptionNet architecture was suggested in [21]. Vilouras implemented CNNs and concluded that two modified Resnet, including “shake–Shake” regularization and squeeze–excitation block, had better accuracy [8].

McDonnell selected a residual network pre-activated CNN and rounded the layer values to reduce memory usage [10]. A modified SegNet [12], fine-resolution CNN (FRCNN) [13] and a multi-scale feature fusion CNN [14] are other types of modified CNNs that have been used for ASC. The generative adversarial neural networks (GAN) [15], CNN with cross-entropy (CE) as loss function [16], CNN including a semantic neighbors over time (SeNoT) module [17], optimized CNNs [18,19,20] and conditional autoencoders [22] are among the deep learning methods used for audio scene classification. All of the above research has a similar feature: the use of log-Mel spectrogram. A one-dimensional, CNN, comprised of multiple convolutional/pooling layers followed by fully-connected layers, was suggested in [24]. A modified 2D CNN, long short-term memory (LSTM) and VGG16 CNN with a processed log-Mel spectrogram were suggested in [25,26,27], respectively.

An encoder–decoder network [27] and Multi-kernel CNN-DNN architecture [28] with log-Mel spectrogram, gamma and CQT are other suggested CNN based classification schemes. Three modified CNN [30,32,33], Resnet based CNN [29] and Four-pathway residual CNNs [31], which use log Mel spectrograms, delta and delta–delta features, are other proposed classification methods. Optimized CNN [4], Fully CNN [34], Light CNN (LCNN) [35], DNN [36,56], VGG16 based CNN [37], SoundNet [39] and Front-end DNN + SVM [40] are among different classification schemes that have been used with hybrid features or RAW data in ASC. Based on the above review, one can conclude that many CNNs, DNNs and other deep learning classification methods have been suggested for ASC. Only a few recent research works have used ensemble learning, SVM, Fuzzy C-means clustering, Adaptive Neuro-Fuzzy Inference System and Naïve–Bayes, as classification schemes. Their need for many training samples, sensitivity to learning parameters, execution time and memory usage are among the main deep learning problems. According to these challenges, a hybrid ensemble learning based method is suggested that overcomes the state-of-the-art deep learning methods. The suggested scheme is trained with fewer samples than the required by the traditional deep learning methods. In the meantime, the sensitivity of the suggested method to training parameters, training time and memory usage is lower than that of the common deep learning based methods.

## 3. Wavelet Scattering

**Figure 1:**
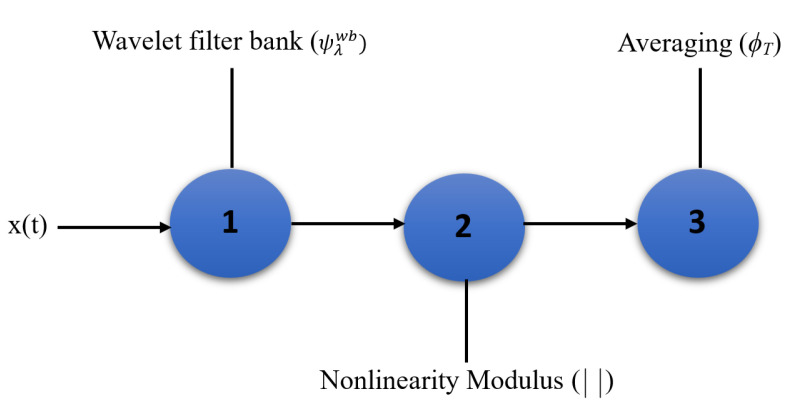
Main steps of WST, where x(t) is the input signal, $\psi_{\lambda }^{wb}$ the wavelet filter bank in λ centre frequency, and $\phi_{T}$ is the averaging in window with width *T*.

In MFC, high-frequency spectrogram coefficients are not stable to time-warping, which means that two signals have a similar form but vary in speed. The MFCC scheme stabilizes these coefficients by averaging them along with Mel frequency. The averaging process dictates some loss of information. A scattering transform recovers the MFCC lost information in averaging with a cascade of wavelet decompositions and modulus operators [57]. It is stable to time warping deformation and locally translation invariant. WST, introduced in [57,58], is an accurate representation based on the iterative wavelet transform modulus. It has been applied to different signal classification tasks, such as synthetic aperture radar [59], speaker identification [60] and ASC [38]. The three main steps of WST are: wavelet filter bank, modulus, and averaging, as depicted in Figure 1.

### 3.1. Wavelet Filter Bank

In wavelet scattering, filter banks are used to assure important properties that are essential to the implementation of WST. These properties include reconstruction and orthogonality as normal wavelet properties and special passband to satisfy the WST concept. First, for signal $x\left(t\right)$, the Fourier transform $X\left(\omega \right)$ is defined as:

(1) $$X\left(\omega \right)=\int_{R}{x(t)e}^{-i\omega t}dt,$$

where *R* is the total time duration of *x*, *i* the imaginary unit, and ω the angular frequency, respectively. An analytic mother wavelet, i.e., Gammatone function, was chosen as a bandpass complex filter ($\psi \left(t\right)$). The Gammatone function is a sinusoid function modulated by a gamma distribution function [61]:

(2) $$\psi \left(t\right)=t^{\left(N-1\right)}e^{-st}u\left(t\right),\;\;\;\;\;\;\;\;\;\;\;\;\;\;\;\;\;\;\;\;\;\;\;\;\;\;\;\;\;\;\;\;\;\;\;s=\alpha -i\omega_{c},$$

where *t* is the time, α the effective duration of ψ, i.e., the filter bandwidth, $\omega_{c}$ the filter centre frequency, $u\left(t\right)$ denotes the unit step function, and *N* is the filter order that determines the transition bands of the filter. In acoustic applications, the Gammatone filter order, typically settled in the range of [3…5], provides a good approximation to the human ear. In this research, N in the first wavelet bank was set to 4 based on [38,62]. It can be easily proved that the Fourier transform of $\psi \left(t\right)$ is [63,64]:

(3) $$\Psi \left(\omega \right)=\frac{(N-1)!}{{\left(\alpha +j\left(\omega -\omega_{c}\right)\right)}^{N}}.\;\;\;\;$$

The suggested filter bank should remove all the signal DC components because these components have no data. In (3), the signal does not have this property, so the Gammatone function’s derivative that introduces a zero at ω=0 is suggested as filter bank functions. The Fourier transform of the time derivative of Gammatone function is:

(4) $$\Psi^{\prime }\left(\omega \right)=j\omega \frac{(N-1)!}{{\left(\alpha +j\left(\omega -\omega_{c}\right)\right)}^{N}}.\;\;\;\;$$

It can be seen that $\Psi^{\prime }\left(0\right)=0$. Hence, the corresponding form of the filter in time domain is:

(5) $$\begin{array}{c} \psi^{\prime }\left(t\right)=\left(N-1\right)t^{\left(N-2\right)}e^{-st}u\left(t\right)\;\;\;-st^{\left(N-1\right)}e^{-st}u\left(t\right)\;\;\\ \;\;\;\;\;\;\;\;\;\;\;\;\;\;\;\;\;=\;\;\;\;\;\left(\;\left(N-1\right)-st\right)t^{\left(N-2\right)}e^{-st}u\left(t\right),\;\;\;\;\;\;\;\\ s=\alpha -i\omega_{c}.\;\;\;\;\;\;\;\;\; \end{array}$$

This form can be used to define a bank of bandpass filters. For simplicity, it is used the following notations:

(6) $$\begin{array}{c} \Psi_{\lambda }^{wb}\left(\omega \right)=\Psi^{\prime }\left(\omega \right),\\ \psi_{\lambda }^{wb}\left(t\right)=\psi^{\prime }\left(t\right), \end{array}$$

where $\psi_{\lambda }^{wb}\left(t\right)$ and $\Psi_{\lambda }^{wb}\left(\omega \right)$ are the time and Fourier transform of the wavelet filter bank with $\lambda =\omega_{c}$ centre frequency.

**Figure 2:**
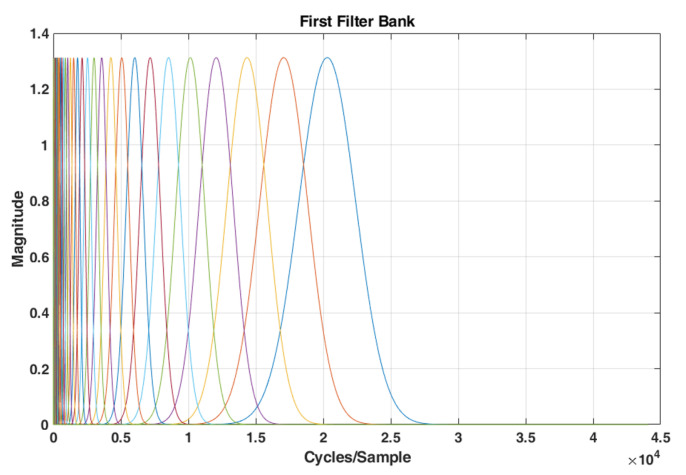
The frequency response of a typical wavelet filter bank with N = 47.

Slaney [65] described each $\omega_{c}$ filter center frequency as:

(7) $$\begin{array}{c} \omega_{c}\left(k\right)=2\pi \left(-C+e^{\frac{k}{K}\;log\left(\frac{f_{\min}+c}{f_{\max}+c}\right)}\cdot \left(f_{max}+C\right)\right), \end{array}$$

subject to:

$$\begin{array}{c} \log_{2}\;\left(\frac{f_{\min}}{f_{\max}}\right)>\frac{1}{NV}, \end{array}$$

where 1≤k≤K, K is the total number of bank filters, *C* is a constant, $f_{\min}$ and $f_{\max}$ are lowest and highest pass band frequencies of the filterbank, and NV is the Number of Voices per octave. The value of $f_{\max}$ must be less than or equal to half of the sampling frequency. The lowest-frequency interval is covered with about equally-spaced filters with constant frequency bandwidth to guarantee that the filter bank covers all frequencies. For simplicity, these filters are still identified as wavelets. The frequency response of filters is given in Figure 2. The narrow band of filters in lower Cycles/Sample shows the inherent property of WST to extract more details in low bands.

### 3.2. Nonlinearity Modulus

**Figure 3:**
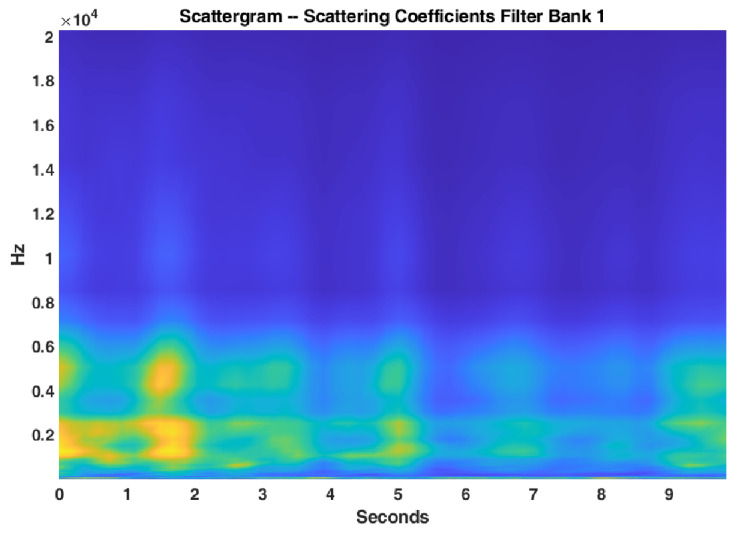
Scalogram of an acoustic scene taking into account the wavelet filter bank of Figure 2.

The output of wavelet filter banks can be calculated by

(8) $$y\left(t,log\lambda \right)=x\left(t\right)*\psi_{\lambda }^{wb}\left(t\right),\;\;\;\;\;\;\;\;\;\;\;\;\;\;\;\;\;\;\;\;\;\;\;\;\;\;\;\;\;\;\;\;\;\;\;\;\;\;\;\;\;\;\;\;for\;\;\;all\;\;\;\;\;\lambda \in \Lambda ,$$

where $x\left(t\right)$ is the input signal, $\psi_{\lambda }^{wb}\left(t\right)$ is defined as in Equation (6), ∗ represents the convolution operator, and the set of all filter bank centre frequencies is denoted by Λ. The modulus of filters is defined by:

(9) $$x_{1}\left(t,log\lambda \right)=\left|y(t,log\lambda )\right|,\;\;\;\;\;\;\;\;\;\;\;\;\;\;\;\;\;\;\;\;\;\;\;\;\;\;\;\;\;\;\;\;\;\;\;\;\;\;\;\;\;\;\;for\;\;\;all\;\;\;\;\;\lambda \in \Lambda ,$$

where || is the modulus, i.e., amplitude, of a complex number, which is the wavelet scalogram. $x_{1}\left(t,\log\lambda \right)$ is a 2D time-frequency representation of the filter bank output, and the wavelet scalogram is a visual representation of this output, which depicts the intensity of $x\left(t\right)$ at time t and λ log frequency. The scalogram of an acoustic scene for wavelet filter bank of Figure 2 is shown in Figure 3.

The scalogram is not time shift-invariant and does not have stability properties. To achieve this, the last step of Figure 1, i.e., averaging step, is necessary.

### 3.3. Averaging

Three operators have been used for making a time shift invariant system in the last step of Figure 1. $x_{1}\left(t,\log\lambda \right)$ is passed from a $\phi_{T}$ low-pass filter, which applies a time averaging to the lowest-frequency interval filters, as:

(10) $$S_{1}x_{1}\left(t,log\lambda \right)=<x_{1}\left(t,log\lambda \right){>}_{t}=x_{1}\left(t,log\lambda \right)*\phi_{T}\left(t\right),$$

where $<\;\;{>}_{t}$ is a notation for time averaging using $\phi_{T}$ and ∗ denotes the convolution integral. The $S_{1}$ output is approximately equal to MFCC, and is commonly known as first order scattering coefficients [58]. This averaging eliminates some high frequencies details. To recover high frequencies details, $x_{1}\left(t,\log\lambda \right)$ is applied to the second wavelet filter bank $\psi_{\gamma }^{wb}\left(t\right)$. Similarly to Equations (8)–(10), making some assumptions, one can have:

(11) $$x_{2}\left(t,log\lambda ,log\gamma \right)=\left|x_{1}\left(t,log\lambda \right)*\psi_{\gamma }^{wb}\left(t\right)\;\right|,\;\;$$

(12) $$S_{2}x_{2}\left(t,log\lambda ,log\gamma \right)=x_{2}\left(t,log\lambda ,log\gamma \right)*\phi_{T}\left(t\right).$$

The second filter bank centre frequencies must be different from the first filter bank. The $\phi_{T}$ low-pass filter is similar in Equations (10) and (12). $S_{2}$ is known as the second-order time scattering coefficients. The final feature vector is a combination of $S_{1}$ and $S_{2}$:

(13) $$Sx=[S_{1}x_{1}\left(t,log\lambda \right),\;\;\;\;\;\;\;\;\;\;\;\;\;\;S_{2}x_{2}\left(t,log\lambda ,log\gamma \right)].$$

Although the above formulas are continuous in time and frequency, the *t*, λ, and γ parameters can be discretized without considerable loss.

## 4. Proposed Method

### 4.1. Training Scheme

**Figure 4:**
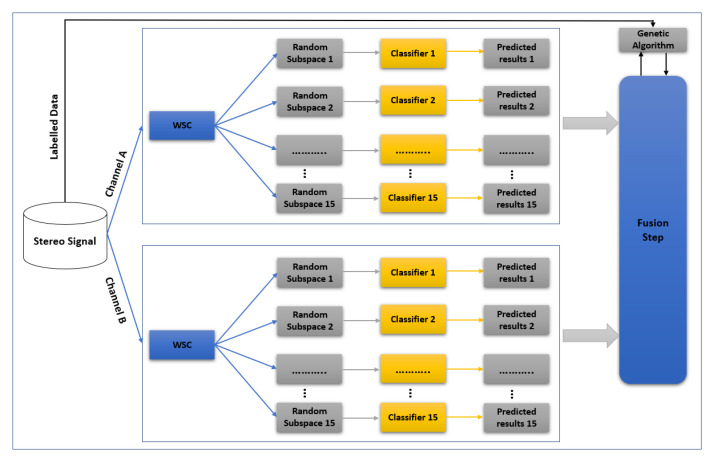
Block diagram of the training scheme of the proposed method.

Figure 4 shows the block diagram of the training scheme of the proposed method. The two channels of input stereo signal are first decomposed to left (A) and right (B) channels. Feature extraction is then performed by applying wavelet scattering to A and B channels separately. Two ensemble classifiers are trained for the channels. The differences, such as delays and noises, between channels lead to different ensemble classifiers that are important in the proposed step. The output of each classifier (A and B) is sent to the fusion step. In the training phase of the fusion step, a genetic algorithm (GA) finds the coefficients of a nonlinear transform to maximize the accuracy of the final result. In fact, GA creates a combination of the two classifier outputs (after their training process) nonlinearly in order to increase the final accuracy. The proposed training scheme was implemented in the Matlab R2020b software package.

#### 4.1.1. Feature Extraction

**Figure 5:**
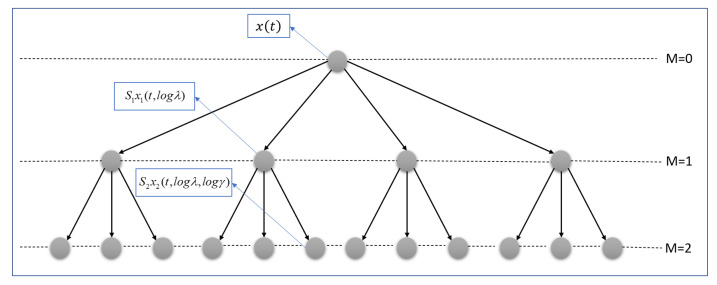
Hierarchical form of WST at first and second filter banks of the proposed method.

WST has been used for feature extraction as a good representation of acoustic scenes (Figure 5). The N term, i.e., the order of the wavelet filter or quality factor, presented in Equations (4) and (5)), in the first wavelet bank (M = 1 in Figure 5) was set to 4, and in the second wavelet filter bank (M = 2 in Figure 5) was set to 1 (one). The invariance scale was chosen equal to 0.75 s. Using these parameters, the number of filter banks in the first step (N = 4) was 47, and in the second step (N = 1) was 13. The highest and lowest filter bank centre frequencies of the first step were 20296 and 3.7 Hz, and for second filter bank were 16,537.5 and 4 Hz, respectively. In a 10 s sound signal with Fs = 44,100 Hz, the size of the extracted feature is 290 × 54, where 290 is the number of resolutions across all orders of the scattering transform, and 54 is the resolution of the scattering coefficients. Quality factors and invariance scale values were chosen based on [23,38].

#### 4.1.2. Ensemble Classifiers

For the classification step, the random subspace method was selected. The random subspace method also called attribute bagging, i.e., feature bagging, is a modified ensemble classifier. Ensemble learning methods typically combine several weak learners in order to make a classifier that works better than the original learners. The random subspace method is an ensemble learning method except that it only uses some training features, which are randomly selected from the training feature set, and are changed for each learner. The random subspace method scheme trains individual learners without over focusing on highly predictive features. For this reason, random subspaces are known as a good choice for acoustic scene classification problems, mainly where the number of features is larger than the number of training data. The random subspace method can be implemented via parallel learning, so it is suitable for fast learning, which is desirable in ASC. The suggested classifier is a systematic construction of a decision forest that relies on a pseudorandom procedure to select each weak learner’s training features. Each weak learner, i.e., decision-tree, generalizes classification by invariances in the excluded features [66]. Finally, the results of the weak learners are combined by averaging the posterior probabilities.

#### 4.1.3. Fusion Step

The proposed fusion step uses two ensemble classifiers output and decides about the scene class. For the fusion step, a nonlinear transform is proposed that must satisfy the following conditions:

- If the result of the classifiers are similar, the value of Fusion result leads to the maximum in this result;
- If the result of the classifiers are different, Fusion result should be maximized in a true class.

To fulfil the above conditions, the following structure was adopted:

- The summation of each class probabilities in two classifiers;
- The summation of class probabilities square in two classifiers;
- The multiply of class probabilities in two classifiers;
- The absolute value of the difference of each class probabilities in two classifiers.

The mathematical formulation of this weighted nonlinear function is:

(14) $$Fusion\;\;\;Result_{i}=\alpha \left(x_{i,A}+x_{i,B}\right)+\beta \left(x_{i,A}^{2}+x_{i,B}^{2}\right)+\gamma \left(x_{i,A}\times x_{i,B}\right)+\lambda \left|x_{i,A}-x_{i,B}\right|$$

where $x_{i,A}$ and $x_{i,B}$ are scores assigned using ensemble classifiers to class *i*, belong to A and B channels, respectively. Obviously, if the result of the classifiers are similar, the value of Equation (14) leads to the maximum and α, β, γ, λ are the parameters that should be found to maximize the accuracy in cases where the classifiers’ results are different.

A genetic algorithm was used to find the best values of α, β, γ, and λ parameters in order to maximize the accuracy of the train set. To avoid unreal answers, the GA search space for α, β, and γ were limited between $\left[0\;\ldots \;\;3\right]$. The true value of λ should be negative because the difference between classifier scores negatively affects the true class. In other words, the classifier scores in true scene should be approximately similar, and the absolute scores’ difference should be minimized. Therefore, the search space for λ was limited between $\left[-3\;\ldots \;\;0\right]$. The optimization toolbox from Matlab software was used for implementing GA, and the final values of the parameters were obtained as: α=1.6406,β=2.8792,γ=1.8059, and λ=−1.5274.

### 4.2. Test Scheme

**Figure 6:**
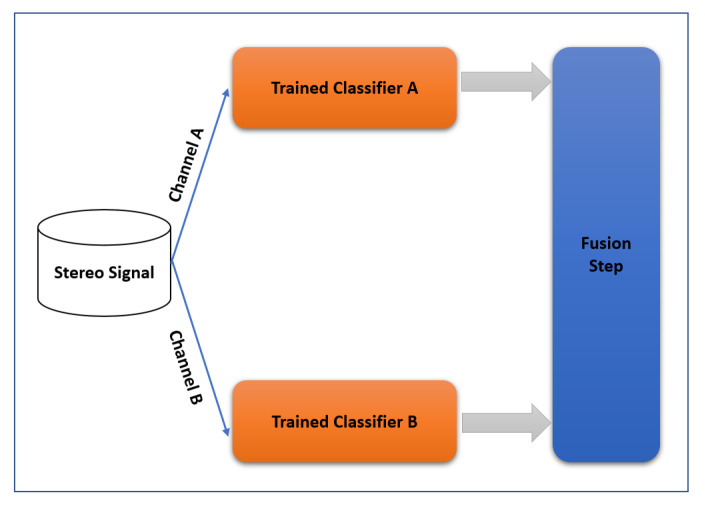
Block diagram of the test scheme of the proposed method.

In the test scheme of the proposed method, Figure 6, the extracted WST features from A and B channels, are applied to train the classifiers. The output of each ensemble classifier, which includes a vector of scores assigned to each class, is used as input of the fusion step. The fusion step calculates (Equation (14)) and chooses the maximum value as final output.

### 4.3. Evaluation Methodology

A dataset from the TUT Acoustic Scenes 2017 (DCASE 2017 challenge) [67] was chosen for the evaluation of the proposed method. This database is a free publicly accessible database, and has been used in most ASC recent research [1,23,27,68,69,70,71,72,73,74,75,76,77,78,79,80,81,82]. The TUT Acoustic Scenes 2017 dataset consists of two subsets: development set and evaluation set, Table 1. The development dataset that is used for the training phase consists of the complete TUT Acoustic Scenes 2016 dataset [83]. The recorded data were divided into subsets based on the original recordings’ location, so the DCASE 2017 evaluation set contains recordings of similar scenes, but from different locations. For each scene, there are 312 audio segments with equal length (10 s). This dataset includes 15 different well balanced classes.

As aforementioned, the TUT Acoustic Scenes 2017 dataset contains 15 different, well balanced, classes:

Bus-travelling by bus in the city (vehicle);
Cafe/Restaurant—small cafe/restaurant (indoor);
Car-driving or travelling as a passenger, in the city (vehicle);
City centre (outdoor);
Forest path (outdoor);
Grocery store -medium size grocery store (indoor);
Home (indoor);
Lakeside beach (outdoor);
Library (indoor);
Metro station (indoor);
Office-multiple persons, typical workday (indoor);
Residential area (outdoor);
Train (travelling, vehicle);
Tram (travelling, vehicle);
Urban park (outdoor).

The proposed method was trained using the development dataset and tested on the evaluation dataset. As in many other works, the confusion matrix was considered an interesting criterion for comparing the proposed method with the existing state-of-the-art methods. As another evaluation criterion, the total accuracy of the proposed method was compared to the ones achieved by some related methods. The total accuracy was calculated by dividing the number of true classifications to the number of total test data.

## 5. Results and Discussion

### 5.1. Results of Ensemble Classifiers and of the Proposed Method

**Figure 7:**
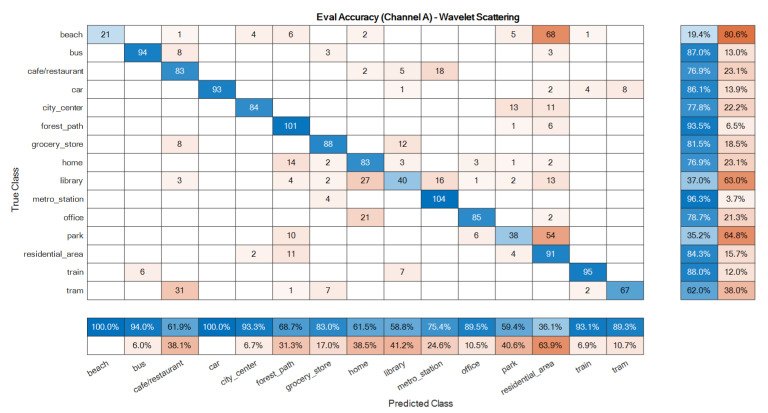
Ensemble classifier confusion matrix for the evaluation data: A channel.

**Figure 8:**
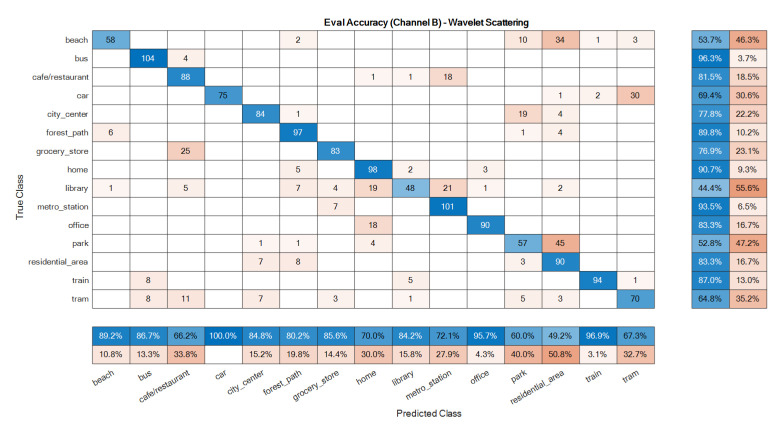
Ensemble classifier confusion matrix for the evaluation data: B channel.

**Figure 9:**
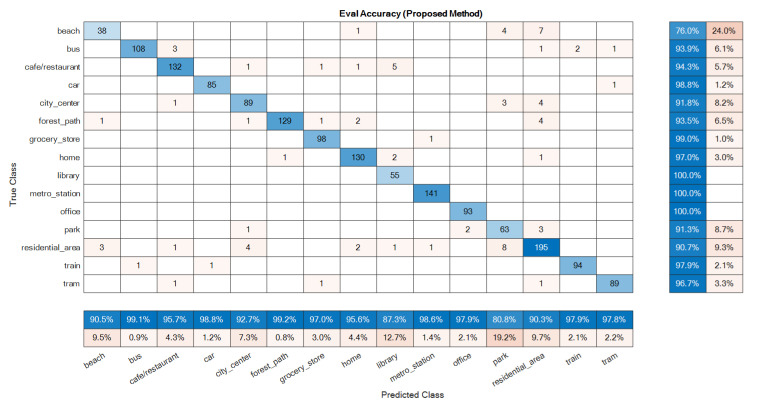
Confusion matrix built for the proposed method as to the evaluation data: stereophonic.

The confusion matrices of the ensemble classifiers separately are given in Figure 7 and Figure 8. The total system confusion matrix, i.e., as to the result after the fusion step, is given in Figure 9. All studied methods showed good results as to the development data. A good model has good accuracy in all classes at the evaluation phase, so these matrices were reported here for the evaluation dataset. The suggested mono ensemble classifiers are fully aligned with Alamir [23] results, which confirms its correct implementation. The differences between mono channels cause differences between ensemble classifiers, Figure 7 and Figure 8, which are relevant in the fusion step. Figure 9 presents the result after the fusion step for the evaluation dataset that shows the correctness of the fusion step of the proposed method. The values in the main diagonals of the matrices in Figure 7, Figure 8 and Figure 9 are the number of correct classifications in the class that belong to that row or column. The other elements are false classifications. In Figure 7, Figure 8 and Figure 9, the main diagonals (correct classifications) are identified by blue, while other elements (false classifications) are in orange. Bolder cells have higher values. (For more details of used colours in Figure 7, Figure 8 and Figure 9, the reader is suggested to see the web version of [23]). The results in Figure 9 confirm the efficiency of the novel fusion step. The main improvements of the novel fusion step are regarding the “residential area” (about 100 false classifications were corrected), “café restaurant” (about 40), “metro station” (about 40), and “home” (about 30 false classifications were corrected) classes, i.e., scenes. In some cases, that one channel was so poor or noisy and the other was good, the fusion step accuracy was lower than of the one of good channel, but intrinsically was better than the one of the poor channel. For example, in the “beach” scene, the number of correct classification that belongs to the A channel was 26 (Figure 7), and, for B channel, it was 58. This difference shows that, in this case, A channel was noisy, and B channel was good. The result after the fusion step was 38, which indicates that the proposed fusion step increased the accuracy of the poor channel, but because the information of both channels was used simultaneously in the fusion step, the accuracy of the results in this class was lower than the one of the good channel.

### 5.2. Sensitivity Analysis

**Figure 10:**
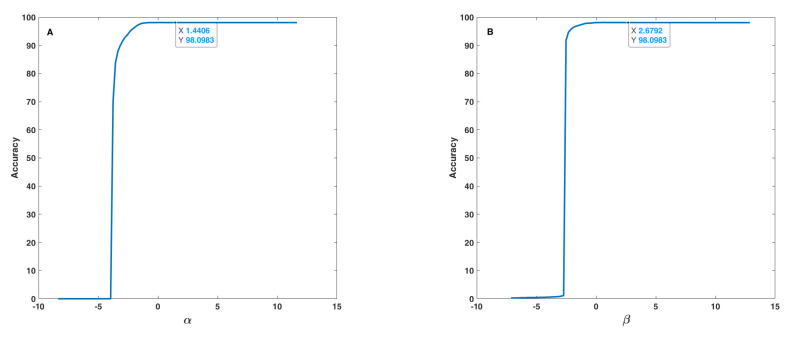
Accuracy in terms of α (**A**) and β (**B**) parameters used in the fusion step (Equation (14)).

**Figure 11:**
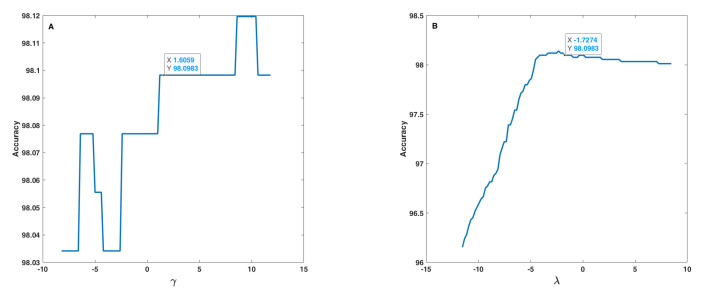
Accuracy in terms of γ (**A**) and λ (**B**) parameters used in the fusion step (Equation (14)).

In the sensitivity analysis of the fusion step, the effect of uncertainty, i.e., of α, β, γ and λ parameters (Equation (14)), on the accuracy of the proposed method was analysed. Because of correlation or mutual effect of these parameters on each other, the 1D and 2D uncertainties were studied. In the 1D sensitivity analysis, one of α, β, γ and λ parameters changed at a time, and the others remain constant. In the 2D analysis, two of α, β, γ and λ parameters change at a time, and the other two remain constant. Therefore, four results for the 1D analysis and six results for the 2D analysis were obtained. Figure 10 and Figure 11 show the results for the 1D analysis. In this analysis, the initial values of the parameters were α = 1.6406, β = 2.8792, γ = 1.8059, and λ = −1.5274 and, in each curve, only one parameter was changed around its initial value. Figure 10 shows the behaviour of accuracy in terms of α and β parameters. These two parameters are very important because both, with some values, lead to an accuracy equal to 0 (zero). α and β shown similar behaviours, and after a threshold value (−4 for α and −2.5 for β), accuracy increased rapidly and reached approximately 100%. The initial point was tagged on each curve according to a safe distance to the threshold points. Figure 11 shows the behaviour of accuracy in terms of γ and λ parameters, which is considerably different relatively to the behaviour found as to α and β parameters. Based on Figure 11, one can realize that γ and λ had a lower effect on accuracy in comparison to α and β and, in fact, these parameters tune the fusion formula efficiently. These observations were under the assumption that the other parameters were fixed in their initial value, but the results indicate that all parameters after a threshold value have no effect on accuracy. On the (A) curve, due to accuracy value, the tagged point was placed in a good interval, and, on the (B) curve, the initial point was placed at a safe distance to the threshold point, and before the accuracy decrease interval.

**Figure 12:**
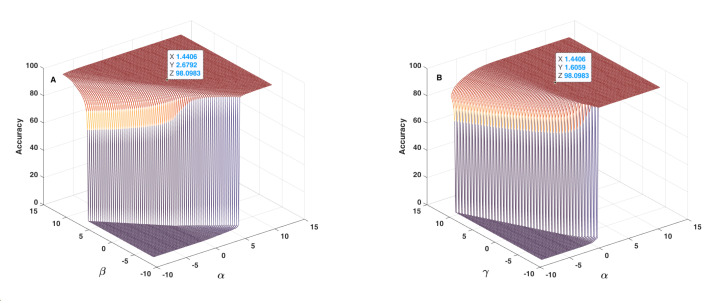
Accuracy in terms of α and β parameters (**A**) and of α and γ parameters (**B**) used in the fusion step (Equation (14)).

**Figure 13:**
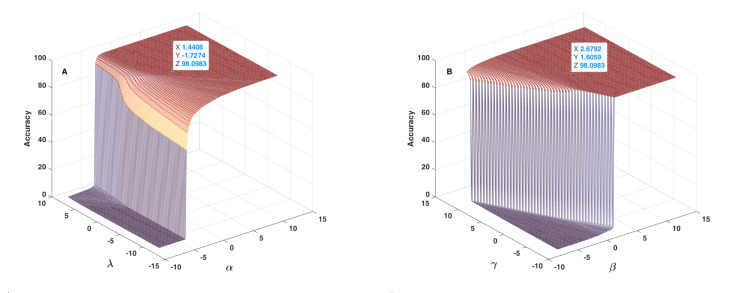
Accuracy in terms of α and λ parameters (**A**) and of β and γ parameters (**B**) used in the fusion step (Equation (14)).

**Figure 14:**
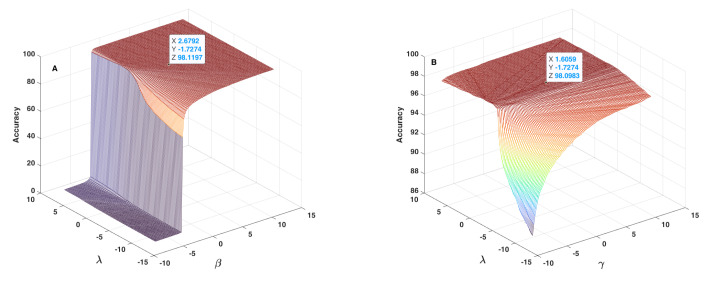
Accuracy in terms of β and λ parameters (**A**) and of λ and γ parameters (**B**) used in the fusion step (Equation (14)).

Figure 12, Figure 13 and Figure 14 show the results obtained as to the 2D sensitivity analysis results. As in the 1D case, the initial values for α, β, γ and λ parameters were equal to 1.6406, 2.8792, 1.8059 and −1.5274, respectively. In each curve, two parameters varied around their initial values and the other two parameters remained constant. Figure 12A shows the behaviour of accuracy in terms of α and β parameters, which have a very similar effect, but based on the 3D plot shown, one can realize that β had higher influence than α. In this figure, the threshold value for changing accuracy from zero to an acceptable value can be defined as a line, between α≈6, β≈−7 and α≈−8, β≈7, which are very different to crisp threshold values found in the case of Figure 10. Figure 12B shows the behaviour of accuracy in terms of α and γ parameters. In each plot of Figure 12, the tagged initial point had a safe distance to the threshold plane where accuracy decreased quickly. Figure 13A shows the behaviour of accuracy in terms of α and λ parameters. The shape of the zero accuracy area is considerably different to the ones of plots of Figure 12, and the threshold value for α in this plot was about −4, which is very similar to the α crisp threshold found for the case of Figure 10A. This similarity suggests that λ has a small effect on α and on accuracy. Figure 13B shows the behaviour of accuracy in terms of β and γ parameters. The observed behaviour is approximately similar to the one depicted in Figure 12B, which indicates the similar effect of α and β parameters. In both plots of Figure 13, the tagged initial point had a safe distance to the threshold plane.

Figure 14A shows the behaviour of accuracy in terms of β and γ parameters, which is similar to the one observed in Figure 13A and confirms once again the similar effect of α and β parameters. Figure 14B lets one conclude that the behaviour of accuracy in terms of λ and γ is different relative to the ones observed for the other cases. In this plot, the lowest accuracy value is around 86%, which indicates that the initial values of α and β parameters keep the accuracy within an acceptable range, and that the effect of λ and γ parameters are lower than the one of α and β parameters. In the meantime, it is possible to conclude that these two parameters can affect accuracy by about 16%, which is considerable and therefore should not be discarded. In addition, in this case, the tagged point had a safe distance to the threshold plane. In conclusion, the sensitivity analysis lets us conclude that the terms including α and β parameters are the main parts of the fusion step, and that the terms including λ and γ parameters can considerably improve the accuracy of the proposed method.

### 5.3. Result Comparison with Previous Studies

The results of the proposed method were compared with the ones obtained by previous studies that also used the TUT Acoustic Scenes 2017 dataset. Usually, the related methods used Mel based features or raw data. The training phase accuracy of all methods under comparison are generally good, but the suggested method showed a considerable improvement in the test phase. Table 2 presents the total accuracy of the methods as to the evaluation dataset obtained under similar conditions. Hence, the methods were trained using TUT Acoustic Scenes 2017 development dataset, and were tested on evaluation of the TUT Acoustic Scenes 2017 dataset. Therefore, the train and test conditions were fully the same for all methods under comparison, and the values as to total accuracy reported in the literature for the related methods were used in Table 2. The accuracy of the proposed method in the training and test phases was 98.1 and 95%, respectively. These results compared to the state-of-the-art methods under comparison indicates that the suggested fusion scheme can be a potential solution for future ASC systems using stereo audio signals data as input.

## 6. Conclusions

In recent years, sound based intelligent systems such as Acoustic scene classification have received a large amount of attention in different applications. This study has presented a two-step method for ASC in stereo signals, which consists of two ensemble classifiers and a novel fusion step. The proposed robust method uses wavelet scattering transform as a stable, time shift invariant transformation. In the proposed method, firstly, two ensemble classifiers were trained using two channels of stereo input. The output of these classifiers was effectively combined using a nonlinear transform to improve the final classification accuracy. The suggestion of a proper, nonlinear transform that satisfies the fusion step conditions and finding the unknown parameters of this transform using a heuristic method, mainly a genetic algorithm, is the main novelty of the proposed method. The classification accuracy obtained using the proposed system in the DCASE 2017 dataset overcome considerably (at least, a 15% of improvement was achieved) the current state of the art as to this well known public dataset. Based on the obtained results, and on its improvement comparing to other ASC methods, the application of the proposed fusion scheme is suggested for other acoustic classification and detection applications such as acoustic event detection.

## Figures and Tables

**Table 1:** Details of the TUT Acoustic Scenes 2017 (DCASE 2017 challenge) ASC dataset.

	Development	Evaluation
**Number of Files**	4680	1620
**Number of Classes**	15	
**Duration per audio signal**	10 s	
**Data Format**	44.1 kHz, 24 bit resolution, Binaural stereo wave files	
**Location**	Dataset was recorded in different cities, including London and Paris.	
**Device**	Roland Edirol R-09 wave recorder	
**Task**	Acoustic Scene Classification	

**Table 2:** Results of different ASC methods and of the proposed ASC method on the evaluation set of the DCASE 2017 dataset.

Ref.	Year	Test Accuracy	Detection Approach
[68]	2017	70	Deep residual CNN
[69]	2017	70.6	DNN
[70]	2017	70.6	CNN
[71]	2017	71.7	Recurrent Neural Network (RNN)
[72]	2017	72.6	CNN
[73]	2017	73.8	CNN
[74]	2017	74.1	CNN
[75]	2017	77.7	DCNN
[76]	2017	80.4	CNN
[77]	2017	83.3	GAN
[78]	2018	64	Deep scalogram representations
[79]	2018	69.9	SVM
[80]	2019	69.3	CNN
[81]	2019	75.4	CNN
[82]	2019	77.1	DCNN
[1]	2020	70	SVM
[23]	2021	80	CNN and Ensemble classifiers
[27]	2021	72.6	DNN
Channel A		72.04	One Ensemble classifier
Channel B		76.36	One Ensemble classifier
Our Method		95	Two Ensemble classifiers and Fusion

## Data Availability

Not applicable.
